# A Phase II Open-Label Trial of Binimetinib and Hydroxychloroquine in Patients With Advanced *KRAS*-Mutant Non-Small Cell Lung Cancer

**DOI:** 10.1093/oncolo/oyad106

**Published:** 2023-04-26

**Authors:** Charu Aggarwal, Alisha P Maity, Joshua M Bauml, Qi Long, Tomas Aleman, Christine Ciunci, Christopher D’Avella, Melissa Volpe, Evan Anderson, Lisa McCormick Jones, Lova Sun, Aditi P Singh, Melina E Marmarelis, Roger B Cohen, Corey J Langer, Ravi Amaravadi

**Affiliations:** Department of Medicine, Division of Hematology-Oncology, University of Pennsylvania, Perelman School of Medicine, Philadelphia, PA, USA; Department of Internal Medicine, Lankenau Medical Center, Wynnewood, PA, USA; Department of Medicine, Division of Hematology-Oncology, University of Pennsylvania, Perelman School of Medicine, Philadelphia, PA, USA; Department of Medicine, Division of Hematology-Oncology, University of Pennsylvania, Perelman School of Medicine, Philadelphia, PA, USA; Department of Medicine, Division of Hematology-Oncology, University of Pennsylvania, Perelman School of Medicine, Philadelphia, PA, USA; Department of Medicine, Division of Hematology-Oncology, University of Pennsylvania, Perelman School of Medicine, Philadelphia, PA, USA; Department of Medicine, Division of Hematology-Oncology, University of Pennsylvania, Perelman School of Medicine, Philadelphia, PA, USA; Department of Medicine, Division of Hematology-Oncology, University of Pennsylvania, Perelman School of Medicine, Philadelphia, PA, USA; Department of Medicine, Division of Hematology-Oncology, University of Pennsylvania, Perelman School of Medicine, Philadelphia, PA, USA; Department of Medicine, Division of Hematology-Oncology, University of Pennsylvania, Perelman School of Medicine, Philadelphia, PA, USA; Department of Medicine, Division of Hematology-Oncology, University of Pennsylvania, Perelman School of Medicine, Philadelphia, PA, USA; Department of Medicine, Division of Hematology-Oncology, University of Pennsylvania, Perelman School of Medicine, Philadelphia, PA, USA; Department of Medicine, Division of Hematology-Oncology, University of Pennsylvania, Perelman School of Medicine, Philadelphia, PA, USA; Department of Medicine, Division of Hematology-Oncology, University of Pennsylvania, Perelman School of Medicine, Philadelphia, PA, USA; Department of Medicine, Division of Hematology-Oncology, University of Pennsylvania, Perelman School of Medicine, Philadelphia, PA, USA; Department of Medicine, Division of Hematology-Oncology, University of Pennsylvania, Perelman School of Medicine, Philadelphia, PA, USA

**Keywords:** non-small cell lung cancer, *KRAS*, MEK 1/2 inhibitor, binimetinib, hydroxychloroquine

## Abstract

**Background:**

In RAS-mutant tumors, combined MEK and autophagy inhibition using chloroquine demonstrated synthetic lethality in preclinical studies. This phase II trial evaluated the safety and activity of the MEK inhibitor binimetinib combined with hydroxychloroquine (HCQ) in patients with advanced *KRAS*-mutant non-small cell lung cancer (NSCLC).

**Methods:**

Eligibility criteria included *KRAS-*mutant NSCLC, progression after first-line therapy, ECOG PS 0-1, and adequate end-organ function. Binimetinib 45 mg was administered orally (p.o.) bid with HCQ 400 mg p.o. bid. The primary endpoint was objective response rate (ORR). A Simon’s 2-stage phase II clinical trial design was used, with an α error of 5% and a power β of 80%, anticipating an ORR of 30% to proceed to the 2-stage expansion.

**Results:**

Between April 2021 and January 2022, 9 patients were enrolled to stage I: median age 64 years, 44.4% females, 78% smokers. The best response was stable disease in one patient (11.1%). The median progression free survival (PFS) was 1.9 months, and median overall survival (OS) was 5.3 months. Overall, 5 patients (55.6%) developed a grade 3 adverse event (AE). The most common grade 3 toxicity was rash (33%). Pre-specified criteria for stopping the trial early due to lack of efficacy were met.

**Conclusion:**

The combination of B + HCQ in second- or later-line treatment of patients with advanced *KRAS-mutant* NSCLC did not show significant antitumor activity. (ClinicalTrials.gov Identifier: NCT04735068).

## Teaser

This phase II trial evaluated the safety and activity of the MEK inhibitor binimetinib combined with hydroxychloroquine in patients with advanced KRAS-mutant non-small cell lung cancer.

**Lessons Learned**

The combination of binimetinib and hydroxychloroquine did not exhibit strong antitumor activity in second- or later-line treatment for patients with *KRAS-*mutant stage IV non-small cell lung cancer.

## Discussion

Alterations in *KRAS* account for the most common mutations in non-squamous non-small cell lung cancer (NSCLC). Despite availability of 2 orally available tyrosine kinase inhibitors (TKIs) that target *KRAS G12C*-mutant NSCLC, response rates are lower than have been seen with other TKIs. More efficacious targeted treatment approaches are needed. Targeting MAPK signaling has been an attractive strategy to target RAS mutant tumors; however, targeting MAPK alone is complicated due to the emergence of resistance pathways such as autophagy, an adaptive response to metabolic and therapeutic stress. We hypothesized that inhibition of autophagy in combination with MAPK inhibition could be a viable approach to treating *KRAS-*mutant lung cancer.

Hydroxychloroquine (HCQ) and its derivatives bind and inhibit palmitoyl protein thioesterase 1, a lysosomal enzyme that regulates lysosomal acidification^1,2^ Inhibition of PPT1 with HCQ deacidifies lysosomes, inhibiting the last step in autophagy. In preclinical models of RAS mutant tumors, combination of a MEK inhibitor with autophagy inhibition using chloroquine or HCQ has demonstrated synergistic antitumor activity.^3-5^ Binimetinib, an orally bioavailable selective and potent mitogen-activated protein (MAP) kinase kinase (MEK1/2) inhibitor, was approved in 2018 by the US Food and Drug Administration (FDA) for treatment of patients with *BRAF*-mutant melanoma. We hypothesized that HCQ could enhance the therapeutic efficacy of binimetinib by inhibiting autophagy, serving as an effective treatment strategy for patients with stage IV NSCLC. Using a Simon 2-stage design, this prospective study regimen was to be considered unworthy of further development if the ORR was ≤30%. Nine patients were enrolled in stage I of this clinical trial, without any observed objective responses. Five of 9 patients (56%) developed a grade >3 AE with rash as the most common grade 3 toxicity (33%; Table 1). No grade 4 treatment-related AEs or treatment-related deaths occurred. Our results show that the combination of B and HCQ does not have significant antitumor activity in ­second-line or beyond treatment of *KRAS-mutant* stage IV NSCLC.

**Table 1.** Adverse events.

AEs	*n* (%)	Grades 1-2, *n* (%)	Grade 3, *n* (%)
Any TRAE	9 (100)	–	–
Grades 1-2 TRAE	9 (100)	–	–
Grades 3-5 TRAE	5 (56)	–	–
Treatment-related SAE	4 (44)	–	–
Treatment related to discontinuation	1 (11)	–	–
Treatment leading to death	0 (0)	–	–
Most common TRAEs (>10% of patients)			
Rash	6 (67)	3 (33)	3 (33)
Diarrhea	5 (56)	4 (44)	1 (11)
Pruritus	3 (33)	3 (33)	0 (0)
Fatigue	3 (33)	3 (33)	0 (0)
Other AEs of interest			
Retinal detachment	1 (11)	0 (0)	1 (11)
Increase in AST	1 (11)	0 (0)	1 (11)
Increase in ALT	1 (11)	1 (11)	0 (0)

Abbreviation: AE: adverse events; AST: aspartate aminotransferase; ALT: alanine aminotransferase; SAE: serious adverse event; TRAE: treatment emergent adverse event.

**Table:** 

Trial Information	
Disease	Lung cancer—NSCLC
Stage of disease/treatment	Metastatic/advanced
Prior therapy	At least one prior regimen
Type of study	Phase II, single arm
Primary endpoint	Objective response rate, safety, and tolerability
Secondary endpoints	Progression-free survival, overall survival
Investigator’s analysis	Inactive because results did not meet primary endpoint
Additional details of study design	This single-arm phase II study enrolled patients with cytologically or histologically confirmed stage IV NSCLC with the presence of a non-synonymous mutation in *KRAS*. The eligibility criteria included age ≥18 years, ECOG PS 0-1, disease progression after at least one prior systemic therapy for metastatic NSCLC, measurable disease based on the RECIST 1.1 criteria, adequate end-organ function, and able to provide written informed consent. Patients with asymptomatic or treated, stable brain metastases were allowed to enrol. All participants provided written informed consent prior to participation. The study was approved by the Institutional Review Board at the University of Pennsylvania and registered at ClinicialTrials.gov (NCT04735068).

**Table:** 

Drug Information	
Generic/working name	Binimetinib
Company name	Pfizer
Drug type	Small molecule
Drug class	MEK 1/2 inhibitor
Dose	45 mg per dose
Route	p.o.
Schedule of administration	45 mg 2 times daily over a 28-day treatment course, until disease progression, unacceptable toxic effects, or withdrawal of consent
Generic/working name	Hydroxychloroquine
Company name	Commercially available
Drug type	Immunosuppressant
Drug class	Immunosuppressant
Dose	400 mg per dose
Route	p.o.
Schedule of administration	400 mg by mouth every 12 hours daily over a 28-day treatment course, until disease progression, unacceptable toxic effects, or withdrawal of consent

**Table:** 

Patient Characteristics	
Number of patients, male	5
Number of patients, female	4
Stage	IV
Age, median (range)	64 (52-77) years
Number of prior systemic therapies: median (range)	3 (2-10)
Performance status: ECOG	0-1: 9 2: 0 3: 0 4: 0
Cancer types or histologic subtypes	Adenocarcinoma, 9

**Table:** 

Primary Assessment method: Objective Response Rate	
Number of patients screened	11
Number of patients enrolled	9
Number of patients evaluable for toxicity	9
Number of patients evaluated for efficacy	9
Evaluation method	RECIST 1.1
Response assessment, CR	0 (0%)
Response assessment, PR	0 (0%)
Response assessment, SD	1 (11.1%)
Response assessment, PD	8 (88.9%)
Median duration assessment, PFS	1.9 months
Median duration assessment, OS	5.3 months

## Assessment, Analysis, and Discussion

**Table:** 

Completion	Study completed
Investigator’s assessment	Inactive because results did not meet primary endpoint

The addition of HCQ to the MEK inhibitor binimetinib did not demonstrate enough activity to justify expansion in a 2-stage phase II trial in patients with *KRAS*-mutant NSCLC. There were no objective responses, and patients had a short median PFS and OS. Further study of binimetinib plus HCQ in patients with metastatic NSCLC is not warranted based on these results.

Despite being the most mutated gene in nonsquamous NSCLC, *KRAS* mutations have been historically very difficult to target therapeutically. Recently, 2 targeted agents, sotorasib and adagrasib, were approved by the FDA for patients with *KRAS G12C*-mutant NSCLC, following progression on first-line therapy for metastatic NSCLC. While these agents are a welcome addition to our armamentarium of targeted therapies, there are currently no viable options for patients with non-G12C *KRAS*-mutant NSCLC. After treatment with platinum doublet-based regimens and a targeted therapy if appropriate, the only available therapy are agents such as docetaxel, which are associated with very poor response rate and survival outcomes with significant toxicity. There is thus an unmet need for better targeted approaches for patients with *KRAS*-mutant NSCLC.

Autophagy is a cellular defense mechanism that has evolved to protect cells from adverse environmental conditions, including nutritional deprivation, hypoxia, or therapeutic stress. Activation of autophagy mediates regulated catabolism of cellular organelles, which are encapsulated first in autophagosomes and metabolized when these fuse to lysosomes. Autophagy has been demonstrated to be a resistance mechanism to chemotherapy in several solid tumors. Inhibition of autophagy can be accomplished pharmacologically with HCQ, which inhibits the fusion of the autophagosome to the lysosome. Several clinical trials have demonstrated the safety of this approach in patients with advanced solid tumors. Pharmacokinetic-pharmacodynamic studies have demonstrated that doses of HCQ 400 mg twice daily or higher effectively blocks autophagy when given alone or in combination with other cancer therapies. In addition, in preclinical models, combination of a MEK inhibitor with HCQ has been shown to be synthetically lethal to RAS mutant cancers. The safety and clinical antitumor activity of combining dabrafenib, trametinib (MEK inhibitor), and HCQ has been demonstrated in patients with *BRAF*-mutant melanoma.^6^ A randomized trial of this combination therapy is being conducted in patients with *BRAFV600*-mutant melanoma with elevated LDH and previous immunotherapy. Together, these data provided the rationale for studying a MEK inhibitor in combination with HCQ in *KRAS*-mutant non-small cell lung cancer.

Nine patients were enrolled onto stage I of this clinical trial. Most patients (77.7%) had received >3 regimens for metastatic disease. Even though all patients had a favorable ECOG PS (0-1) at study entry, most patients had exposure to several lines of previous cytotoxic therapies. Patients on this trial had a variety of molecular alterations, including *KRAS G12V*, *G12D*, and *G12F*.

The combination of HCQ and binimetinib was poorly tolerated. Overall, 5 of 9 patients (55.6%) developed a grade 3 adverse event (AE), with rash being the most common grade 3 toxicity. These toxicities required dose reductions of binimetinib in 2 patients. Most patients (*n* = 7) received <3 cycles of therapy on trial. The median dose intensity of both drugs was 75%. It is unknown whether longer exposure to this combination might increase the incidence and severity of toxicity. The dose and schedule were predetermined based on other phase I/II trials.^7^ We did not perform pharmacokinetic testing and cannot exclude the possibility of a drug-drug interaction. These findings suggest that further pharmacokinetic and pharmacodynamic modeling may be required prior to further study so that toxicity can be balanced with efficacy. Expansion to stage II of the trial was not pursued, per protocol, due to lack of activity. Efficacy analysis of the patients treated on the study shows median PFS (1.9 months) and OS (5.3 months). No objective responses were seen.

Our study has some limitations. First, it was a single-arm study with no randomized control design, and hence, selection bias could not be ruled out. Second, we enrolled a heterogenous patient population with different *KRAS* mutations under the assumption that they would all potentially respond to the studied drug combination. This was based on the preclinical data showing synthetic lethality in RAS mutant cancers regardless of mutation type.

Finally, the dose and schedule were predetermined based on other phase I/II trials, but this might not be the best-suited dose as the next step in patients with NSCLC. Novel autophagy inhibitors such as DCC-3116 (NCT04892017) could be potentially more effective than HCQ, in combination with MEK inhibition in *KRAS-*mutant NSCLC. In conclusion, the current study showed that the combination of binimetinib combined with HCQ in second- or later-line treatment of advanced *KRAS-*mutant NSCLC did not exhibit meaningful antitumor activity and is not worthy of further study.

## Data Availability

The data underlying this article will be shared on reasonable request to the corresponding author.

## References

[1] Rebecca VW , Nicastri MC, McLaughlin N, et al. A unified approach to targeting the Lysosome’s degradative and growth signaling roles. Dimeric quinacrines identify new lysosomal target in cancer. Cancer Discov. 2017;7(11):1266-1283. 10.1158/2159-8290.CD-17-0741 28899863 PMC5833978

[2] Rebecca VW , Nicastri MC, Fennelly C, et al. PPT1 promotes tumor growth and is the molecular target of chloroquine derivatives in cancer. Cancer Discov. 2019;9(2):220-229. 10.1158/2159-8290.CD-18-0706 30442709 PMC6368875

[3] Kinsey CG , Camolotto SA, Boespflug AM, et al. Protective autophagy elicited by RAF→ MEK→ ERK inhibition suggests a treatment strategy for RAS-driven cancers. Nat Med. 2019;25(4):620-627. 10.1038/s41591-019-0367-9 30833748 PMC6452642

[4] Bryant KL , Stalnecker CA, Zeitouni D, et al. Combination of ERK and autophagy inhibition as a treatment approach for pancreatic cancer. Nat Med. 2019;25(4):628-640. 10.1038/s41591-019-0368-8 30833752 PMC6484853

[5] Lee C-S , Lee LC, Yuan TL, et al. MAP kinase and autophagy pathways cooperate to maintain RAS mutant cancer cell survival. Proc Natl Acad Sci USA. 2019;116(10):4508-4517. 10.1073/pnas.1817494116 30709910 PMC6410784

[6] Mehnert JM , Mitchell TC, Huang AC, et al. BAMM (BRAF Autophagy and MEK Inhibition in Melanoma): a phase I/II trial of dabrafenib, trametinib, and hydroxychloroquine in advanced BRAFV600-mutant melanoma. The BAMM trial in BRAF-mutant melanoma. Clin Cancer Res. 2022;28(6):1098-1106. 35022320 10.1158/1078-0432.CCR-21-3382 PMC8923957

[7] Rangwala R , Leone R, Chang YC, et al. Phase I trial of hydroxychloroquine with dose-intense temozolomide in patients with ­advanced solid tumors and melanoma. Autophagy. 2014;10(8):1369-1379. 10.4161/auto.29118 24991839 PMC4203514

